# CoGames: Development of an adaptive smartphone-based and gamified monitoring tool for cognitive function in Multiple Sclerosis

**DOI:** 10.1007/s00415-024-12818-y

**Published:** 2025-01-15

**Authors:** Silvan Pless, Tim Woelfle, Johannes Lorscheider, Andrea Wiencierz, Óscar Reyes, Carlos Luque, Pasquale Calabrese, Cristina Granziera, Ludwig Kappos

**Affiliations:** 1 Research Center for Clinical Neuroimmunology and Neuroscience Basel (RC2NB), University Hospital Basel, University of Basel, Spitalstrasse 2, CH-4031 Basel, Switzerland; 2 Neuropsychology and Behavioral Neurology Unit, Department of Psychology and Interdisciplinary Platform Psychiatry and Psychology, Division of Molecular and Cognitive Neuroscience, University of Basel, Basel, Switzerland; 3 Department of Neurology, University Hospital Basel, Basel, Switzerland; 4 Department of Clinical Research, University Hospital Basel, University of Basel, Basel, Switzerland; 5 Indivi (a DBA of Healios AG), Basel, Switzerland

**Keywords:** Multiple sclerosis, Cognitive assessment, Gamification, Monitoring tool, Smartphone games

## Abstract

**Aim:**

As part of the development of a smartphone-based app for monitoring MS disease activity and progression (dreaMS, NCT05009160), we developed six gamified tests with multiple difficulty levels as a monitoring tool for cognition. This study quantified the relative difficulty between levels and investigated their reliability, ability to depict practice effects, and user acceptance.

**Methods:**

Healthy volunteers played each game, covering five cognitive domains, twice per day for 11 consecutive days. Linear mixed models determined the relative difficulty of the levels. Spearman's correlation of the two daily repetitions measured test–retest reliability. Difficulty increased daily except for days 2, 6, and 11, when the easiest level (“Beginner”) was repeated to estimate practice effects. Participants rated enjoyment and other components of acceptance on a 5-point scale.

**Results:**

We included 82 participants from April to July 2023 in Basel, Switzerland, of which 76 (51 female, age: 40.3 ± 13.9 years, range 18–69) completed the study according to protocol. Generally, mean performances decreased with higher difficulty levels. Across all levels of all games, the median test–retest correlation was 0.825 (range of medians 0.55–0.9). Mean performance in level “Beginner” improved across all games. The mean enjoyment rating was 3.9 (range: 3.1–4.3).

**Conclusion:**

Our study showed that the CoGames yield reliable measures across different cognitive domains and difficulty levels and were enjoyable to play. The observed practice effects must be considered, but also indicate sensitivity to change. These results support the hypothesis that adaptive gamified digital tests can serve as a reliable and well-accepted monitoring tool of cognition in PwMS.

**Supplementary Information:**

The online version contains supplementary material available at 10.1007/s00415-024-12818-y.

## Introduction

In multiple sclerosis (MS), a chronic inflammatory and neurodegenerative autoimmune disease affecting the central nervous system, up to 70% of patients display signs of cognitive impairment (CI), often negatively influencing emotional well-being, quality of life, and working capacity [1–5]. Still, comprehensive monitoring of cognitive functions is not a standard procedure in most clinics [6]. This can mainly be attributed to the fact that such assessments are time-consuming, costly, and often burdensome for patients [6]. Novel assessment tools that are well accepted, convenient, and sensitive to change are needed. Digital devices such as computers, tablets, and smartphones have been used as a basis for medical rehabilitation and assessments for several years [7–16]. Especially smartphones with their wide distribution in the general population, high-quality sensors, and user-friendliness have great potential to contribute to accurate, remote, and unbiased assessment [17].

In parallel to the emerging use of digital devices in the medical field, the inclusion of gamification elements in medical devices has gained popularity as well [9, 10, 15, 18]. Using motivational gamification elements in an assessment tool might improve adherence and motivate patients to reach their maximum performance [19, 20]. For example, a scoring and reward system can elicit feelings of competition and accomplishment [20, 21]. Furthermore, a dynamic difficulty adjustment (DDA) system comprising multiple difficulty levels can prevent frustration and boredom and promote a flow state, a state of high concentration [22, 23].

We hypothesize that well-designed smartphone games might be the optimal tool to circumvent the shortcomings of current established neuropsychological assessments [18, 24]. To further investigate this and as part of the development of a smartphone-based app for monitoring MS disease activity and progression (dreaMS, NCT05009160, BASEC-ID: 2021- D0040), we developed a set of multi-leveled smartphone games to assess cognitive function. As a first step, the CoGames study aimed to investigate the relative difficulty between levels, reliability across difficulty levels, estimate potential practice effects on performance over time, and assess their acceptance.

## Methods

In cooperation with the medical device software manufacturer Indivi Ltd. (a DBA of Healios AG), we developed a set of smartphone games for the assessment of cognitive function [25, 26]. The six games cover five cognitive domains: working memory, information processing speed, short-term memory, psychomotor speed, and mental flexibility (set-shifting between two cognitive tasks). Information processing speed, as the most frequently affected cognitive domain in PwMS, is assessed in two games [27].

### Standard protocol approvals, registrations, and patient consents

The CoGames study was conducted according to the standards of the World Medical Association Declaration of Helsinki and approved by the local ethics committee: Ethikkommission Nordwest- und Zentralschweiz (EKNZ), Basel, Switzerland (Req-2022–01571).

### Study procedures

From April to July 2023, we recruited healthy volunteers via flyers and online advertisements in Basel, Switzerland. The participants were screened and instructed in a phone call. Information regarding the download of the app and the daily schedule was additionally sent by email. Participants were instructed to play the cognitive games autonomously at home on a daily basis for 11 consecutive days. The 11 days include performing all eight difficulty levels with one repetition per day for test–retest analysis and 3 days for completing the easiest level to assess change in performance over time. We decided to use a twice-daily frequency to keep the study duration as short and convenient to the participants as possible. To measure reliability via test–retest correlation, the participants were asked to play the same difficulty level of every game twice per day. Every day a different level was assessed, starting with the easiest and increasing difficulty daily, except for days 2, 6, and 11 where level 1 (“Beginner”) was repeated, to observe potential practice effects. To investigate the relative differences in difficulty between the levels, we applied linear mixed models with user as the random effect and difficulty levels, age, operating system, years of education, and their interaction with difficulty levels as fixed effects. The easiest level “Beginner” was used as the reference (intercept). Furthermore, we established a standardization allowing to compare scores across different difficulty levels. On days 4, 8, and 11 participants were asked to answer a short online questionnaire to assess overall acceptance and the impact of the different difficulty levels on acceptance. An overview of the study schedule is shown in Table 1. After the completion of each game, the data were uploaded and stored on a general data protection regulation (GDPR)-compliant secure cloud (Google Firebase: https://firebase.google.com/docs/storage/). From there, the raw data were preprocessed and sent to the intranet servers of the University Hospital Basel.

**Table 1:** Study schedule

Task/day	0	1	2	3	4	5	6	7	8	9	10	11
Screening	X											
Instructions	X											
Questionnaire					X				X			X
Lvl.1 Beginner		X	X				X					X
Lvl.2 Apprentice				X								
Lvl.3 Graduate					X							
Lvl.4 Expert						X						
Lvl.5 Star								X				
Lvl.6 Superstar									X	*	*	
Lvl.7 Hero										X		
Lvl.8 Deity											X	

^*^Catch-a-Cloud only consists of six difficulty levels, and thus level 6 was repeated until the end of the study

### Participants

Inclusion criteria were informed consent, age 18–80 years, owning and proficiency to use a smartphone, which met the technical requirements of the CoGames app (iOS ≥ 13.0, android ≥ 7.0). Exclusion criteria comprised any current or past neurological or psychiatric disease and any current dexterity or uncorrected vision impairments. Furthermore, participants using any medication or recreational drugs that are known to impact cognitive function or hand–eye coordination were not included.

### Instruments and measurements

**Fig. 1:**
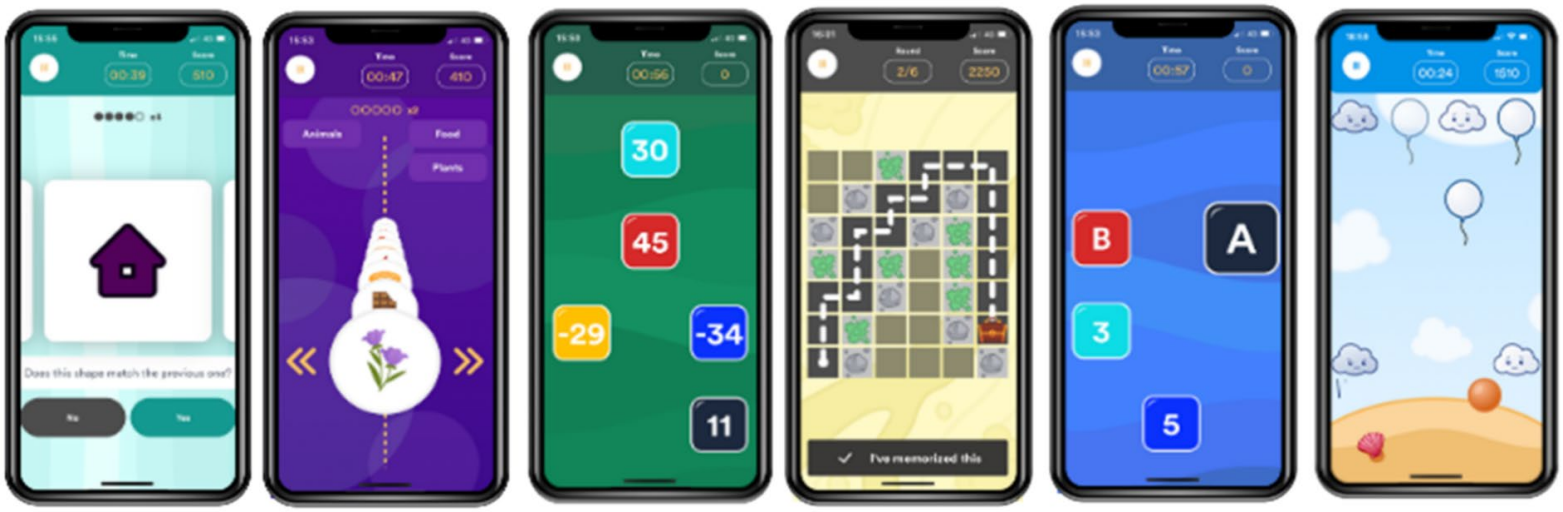
Screenshots of the challenges included in CoGames. *From left to right: Match Maker, Think Fast, Numbers, Treasure Hunt, Mixer, Catch-a-Cloud

All cognitive games comprised eight difficulty levels except for the psychomotor game Catch-a-Cloud, which comprised only six levels. Measures that represent game performance were selected based on established neuropsychological tests: number of correct responses and percentage of correct connections. To keep the games as convenient and enjoyable as possible, we limited the game time to maximum 1 min except for the memory game Treasure Hunt and the psychomotor game Catch-a-Cloud. In Treasure Hunt we allow the participants to memorize and recall for as long as they need. Catch-a-Cloud lasts 50 s and is divided into two phases: 20 s Test Mode followed by 30 s Game Mode. The Test Mode contains fewer gamification elements, while in the Game Mode there are additional gamification elements, changing from level to level. All measures used for the analyses, brief game descriptions, and the cognitive domain they aim to assess are described in Table 2. Screenshots of all games are shown in Fig. 1: screenshots of the challenges included in CoGames. Example videos of all CoGames are included in Online Resource S2-7.

**Table 2:** Domains, measures, and description of CoGames

Cognitive domain	Game (no. of levels)	Primary measure	Description
Working memory	Match maker 8 levels	Number of correct responses in 60 s	The system shows an either colorful or gray shape on the screen. With every answer, a new shape appears and the previous is hidden. The task is to continuously decide (“Yes”/”No”) whether the presented shape matches the one shown “n” taps before. “N” increases with higher difficulty
Information processing speed	Think fast 8 levels	Number of correct responses in 60 s	The system shows an image which must be sorted into the correct category as fast as possible. The number of categories and their similarity change with difficulty
	Numbers 8 levels	Number of correct responses in 60 s	The system displays a series of numbers that are randomly placed across the screen. The participant’s task is to tap the numbers in ascending order, as fast as possible
Short-term memory	Treasure hunt 8 levels	Mean percentage of correct grid–square connections over 6 rounds	The system shows a grid with a path to an “X” (treasure). The participant is asked to memorize the exact path to the treasure. The participant is then asked to reconstruct the path on a blank grid. Grid size, path length, and intermission time (time between memorization and reconstruction phase) are increased with higher difficulty
Mental flexibility	Mixer 8 levels	Number of correct responses in 60 s	The system displays a series of letters and numbers that are randomly placed across the screen. The participant must tap the numbers and letters in ascending/alphabetical order, as fast as possible, always alternating between the two
Psychomotor speed	Catch-a-cloud 6 levels	Number of correct responses in 50 s (Test Mode: 20 s, Game Mode: 30 s)	The system displays clouds on screen which must be “popped” by tapping on them as fast as possible. Once popped, the cloud reappears at another location on the screen. The first 20 s are the Test Mode (TM), which only shows 1 cloud on screen. After the TM, the Game Mode (GM) follows for 30 s. The GM includes gamification elements such as raining clouds, distractions (Balloons) which must be avoided, and multiple clouds at once

The acceptance questionnaire comprised five questions which were to be rated on a 5-point Likert scale (1 = most negative, to 5 = most positive): enjoyment, representation of mental performance, adequacy of difficulty increase, clarity of the instructions, and level of frustration. The full questionnaire is shown in the supplementary material (S1. Acceptance questionnaire).

### Objectives and outcomes

Our objectives were to a) investigate the relative differences between difficulty levels; b) assess reliability; c) estimate potential practice effects and gain first evidence that the CoGames can detect change in performance over time, and d) assess acceptance by participants with a focus on enjoyment. Hence, our outcomes were a) disparities in linear mixed model estimates between levels, corrected for age, operating systems, education, and their interactions with difficulty levels. Each estimated parameter directly represents the difference in the mean performance outcome (e.g., number of correct responses in 60 s) between the level and the reference level. The further the estimated parameter is from zero, the greater is the difference; b) Spearman’s correlation coefficient between two repetitions of the same game and difficulty level, played on the same day. A correlation coefficient of *r* ≥ 0.6 was regarded as acceptable; c) increase of scores across four repetitions of the level “Beginner” as estimate of practice effects and ability to detect change; d) mean ratings over the three rating days on the acceptance questionnaire. A score of ≥ 3 on the 5-point Likert scale was defined as sufficiently accepted by participants.

### Data collection, statistical analyses, data access, and availability statement

Data collection, data cleaning, and feature extraction processes were performed using Python 3.9 coding language. C. L. and Ó. R. take full responsibility of these processes. Statistical analyses were performed using R version 4.4.0 (24.04.2024). L. K., S. P., and A. W. take full responsibility for the data, analyses and interpretation, and the conduct of the research and had full access to all the data and the right to publish any data separate and apart from any sponsor. The data supporting the findings of this study are available from the corresponding author upon reasonable request.

## Results

We recruited 82 participants, 6 of whom dropped out, resulting in a final cohort of 76 participants. Dropouts were caused by technical difficulties (*n* = 3): crashes of the app which led to missing data and non-adherence (*n* = 3); inability to play the games within the fixed time frame foreseen for completion resulting in missing data. In total, a participant was scheduled to complete each game 22 times over 11 days including the 3 repetitions of level 1 (every game 2 × per day). The average overall adherence rate was 90.63%. Table 3 provides an overview of the participants’ demographics.

**Table 3:** Demographics of participants

	Healthy volunteers (*n* = 76)
Female, n (%)	51 (67%)
Mean age ± SD Range	40.3 ± 13.9 years 18–69 years
Adherence (tests completed of total scheduled)	90.63%
Operating system, *n* (%), models	iOS: 44 (58%), 16 different models between iPhone 7 and iPhone14 Android: 32 (42%), 20 different models from Samsung, OnePlus, Oppo, Xiaomi, and Sony
Mean years of education ± SD Range	15 ± 2.5 years 10–20 years

### Outcomes

#### Difficulty level estimation

For all games, the level “Beginner” of day 1 was used as the reference. Detailed outputs of the linear mixed models for every game can be found in the appendix Tables 5, 6, 7, 8, 9, 10, 11. Female and male participants did not show differences in performance. Therefore, we did not include this variable into the linear mixed models. In Match Maker the average number of correct responses in 60 s differed notably between all levels and the reference. Also, participants using iOS performed better (estimate for iOS vs. android: 16.67, 95%-CI [12.45, 20.88], *p* ≤ 0.001), while older participants (estimate: −0.45 per years of age, 95%-CI [−0.59, −0.30], *p* ≤ 0.001) performed worse. In Think Fast, the mean number of correct responses in 60 s of all levels, except for level “Apprentice” was different from the reference. Furthermore, younger age was associated with better scores (estimate: −1.25 per years of age, 95%-CI [−1.51, −0.99], *p* ≤ 0.001). In Numbers, whereas the average number of correct responses in 60 s of the levels “Apprentice” and “Graduate” was not notably different from the reference, the measures of all other levels were. Younger (estimate: −0.23 per years of age, 95%-CI [−0.36, −0.11], *p* ≤ 0.001) and participants with more years of education (estimate: 0.86 per years of education, 95%-CI [0.27, 1.46], p = 0.006) showed higher performance. For Treasure Hunt, only the measures (mean percentage of correct connections) in levels “Apprentice”, “Graduate”, and “Superstar” did not show considerable differences to the reference. In Mixer, the average number of correct responses in 60 s was substantially different between all levels, except for level “Graduate”. Higher performance was also associated with younger age (estimate: −0.29 per years of age, 95%-CI [−0.44, −0.16], *p* ≤ 0.001) and more years of education (estimate: 0.71 per years of education, 95%-CI [0.04, 1.39], *p* ≤ 0.001,). In Catch-a-Cloud Test Mode the average number of correct responses in 20 s did not differ strongly between levels. Younger participants (estimate: −0.24 per years of age, 95%-CI [−0.31, −0.17], *p* ≤ 0.001,) and iOS users (estimate: 5.13, 95%-CI [3.12, 7.10], *p* ≤ 0.001) performed better. For the Catch-a-Cloud Game Mode the average number of correct responses in 30 s of level “Superstar” clearly differed from the reference. Performance was better in younger (estimate: −0.33 per years of age, 95%-CI [−0.43, −0.23], *p* ≤ 0.001,) and iOS users (estimate: 7.70, 95%-CI [5.46, 9.95], *p* ≤ 0.001).

#### Reliability testing: test–retest correlation of the daily repetitions

Sample sizes for reliability calculations vary slightly due to single missing repetitions. The exact sample sizes used for each day are shown in detailed tables in the appendix (Tables 12, 13, 14, 15, 16, 17): Achieved scores and rho (test–retest reliability) by study day and difficulty level. The three easiest levels (“Beginner”, “Apprentice”, and “Graduate”) of the game Treasure Hunt showed strong ceiling effects (see Table 15: Treasure Hunt) and did not reach the target correlation coefficient of |*r*_*s*_* |*≥ 0.6. All other difficulty levels of all games showed significant strong Spearman’s correlation coefficients (|*r*_*s*_* |*= 0.64 – 0.94, *p* ≤ 0.001) in the comparison of test and retest, thus meeting the predefined target of |*r*_*s*_* |*≥ 0.6: Match Maker: Med: 0.88, IQR: 0.77 – 0.91, range: 0.73–0.93; Think Fast: Med: 0.83, IQR: 0.8–0.91, range: 0.74–0.92; Numbers: Med: 0.78, IQR: 0.72–0.81, range: 0.66–0.85; Mixer: Med: 0.82, IQR: 0.78–0.87, range: 0.76–0.92; Catch-a-Cloud: Med: 0.9, IQR: 0.86–0.93, range = 0.84 –0.94. Treasure Hunt, including the levels that did not reach the target: Med: 0.55, QR: 0.32–0.72, range: 0.21–0.74. An overview of the correlation coefficients is shown in Table 4.

**Table 4:** Cognitive domains, primary measures, reliability, and acceptance (enjoyment)

Cognitive domain	Game	Primary measure	Reliability* (Spearman’s rho) Median, IQR Range	Mean enjoyment rating (5-point Likert scale)
Working memory	Match maker	Number of correct responses	0.88 0.77–0.91 0.73–0.93	3.8 (± 0.96)
Processing speed	Think fast	Number of correct responses	0.83 0.8–0.91 0.74–0.92	4.28 (± 0.74)
	Numbers	Number of correct responses	0.78 0.72–0.81 0.66–0.85	4.14 (± 0.85)
Short-term memory	Treasure hunt	Mean percentage of correct connections	0.55 0.32–0.72 0.21–0.74	3.13 (± 1.19)
Mental flexibility	Mixer	Number of correct responses	0.82 0.78–0.87 0.76–0.92	3.74 (± 1.01)
Psychomotor speed	Catch-a-cloud	Number of correct responses	0.9 0.86–0.93 0.84–0.94	4.22 (± 1)

^*^Correlation coefficients include all difficulty levels

All *p* values of the correlations were ≤ 0.001 for all except the repetitions of level 1 (“Beginner”) in Treasure Hunt

#### Change in performance over time

**Fig. 2:**
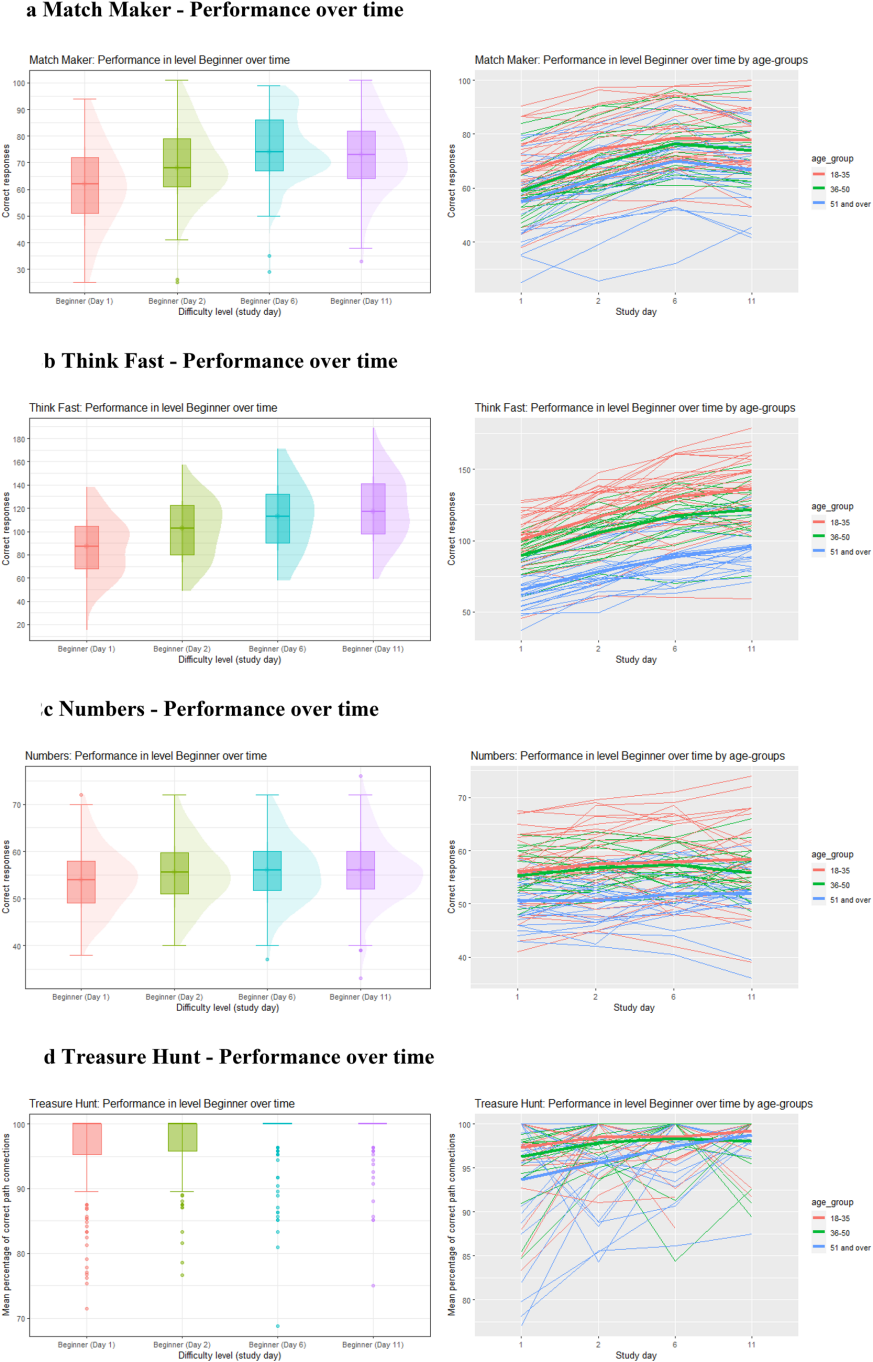

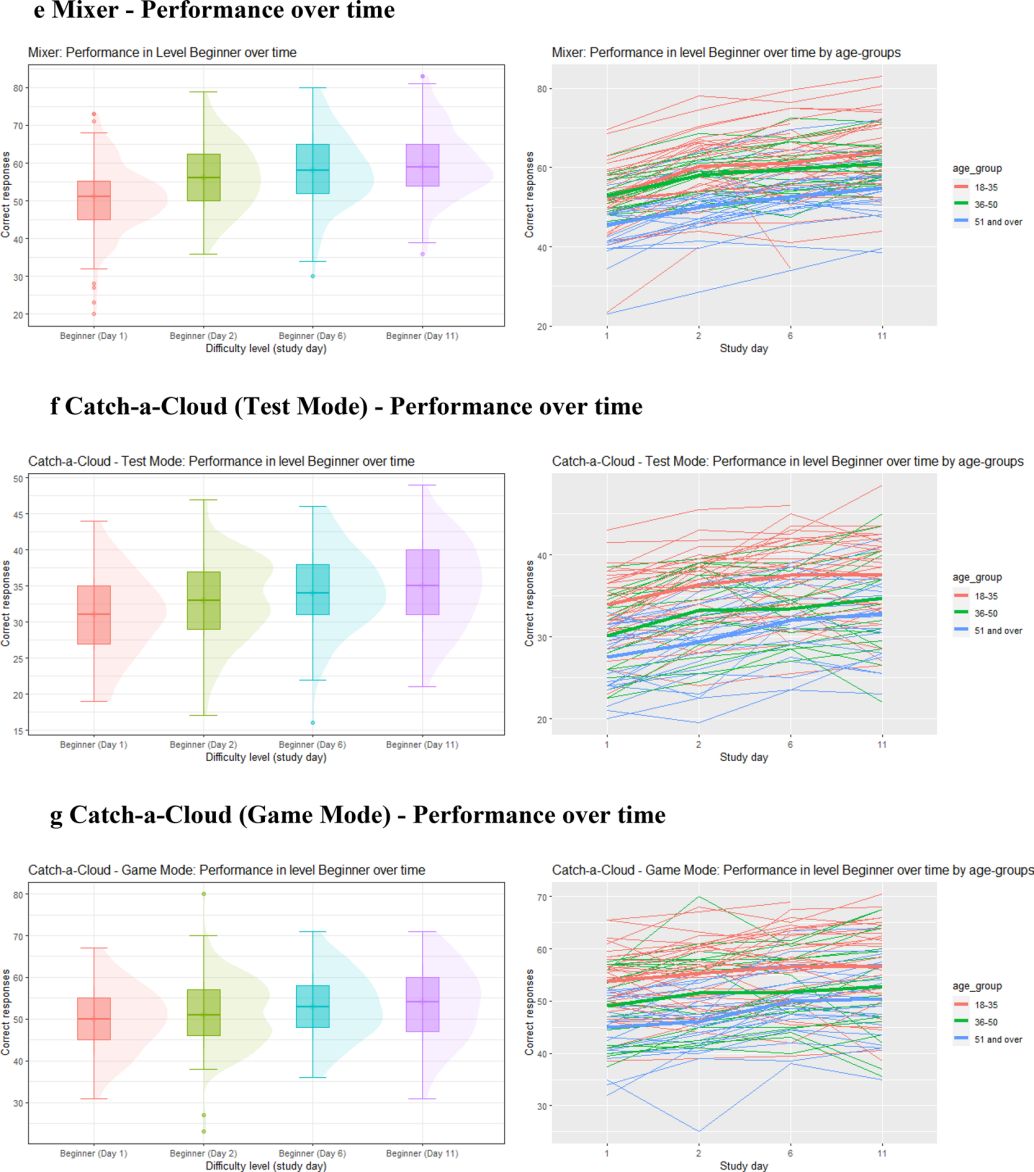
**a** Match Maker: performance over time. **b** Think Fast: performance over time. **c** Numbers: performance over time. **d** Treasure Hunt: performance over time. **e** Mixer: performance over time. **f** Catch-a-Cloud (Test Mode): performance over time. **g** Catch-a-Cloud (Game Mode): performance over time

For most games, there were gradual improvements over time when comparing the mean scores of level 1 (“Beginner”) that was completed on days 1, 2, 6, and 11. In Match Maker the mean score increased from 62 to 69, to 75 and then decreased to 73 on the last day. In Think Fast the score gradually increased from 86 to 103, to 112 to 116. The Numbers scores changed from 54 to 55 to 56 and remained at 56 for the last repetition. For Treasure Hunt the scores increased gradually from 96 to 97, to 98 to 99. In Mixer the scores increased gradually from 51 to 56, to 59 to 60. Lastly, Catch-a-Cloud Beginner scores gradually increased from 50 to 51 to 53 where it stagnated for the last repetition. Details of the results can be found in Figs. 2a–g and the appendix (Tables 12, 13, 14, 15, 16, 17).

#### Acceptance: Enjoyment

The average rating of enjoyment over all rating time points and across all games was 3.8 (range: 3.13–4.29) on a 5-point Likert scale: Match Maker: 3.8 (± 0.96), Think Fast: 4.28 (± 0.74), Numbers: 4.14 (± 0.85), Treasure Hunt: 3.13 (± 1.19), Mixer: 3.74 (± 1.01), Catch-a-Cloud: 4.22 (± 1). All games reached the predefined cutoff of 3 points on the 5-point Likert scale. Clear changes in ratings over time (> 0.5 points) were only found in Treasure Hunt: day 4: 3.47 (± 1.15), day 8: 2.78 (± 1.23), and day 11: 3.13 (± 1.19). An overview of the mean enjoyment ratings of all games is shown in Table 4.

#### Acceptance: representation of cognitive function, perceived difficulty increase, clearness of instructions, and frustration

Across all games, the mean rating on whether the participants find that game performance represents their cognitive abilities was 3.73 (range: 3.26—3.94): Match Maker: 3.88 (± 0.92), Think Fast: 3.94 (± 0.83), Numbers: 3.83 (± 0.92), Treasure Hunt: 3.6 (± 1.15), Mixer: 3.87 (± 0.97), Catch-a-Cloud: 3.26 (± 1.3). There were no strong changes between the three rating days. On average, the participants rated the linearity of difficulty increase as 4 (range: 3.22–4.24): Match Maker was 4.23 (± 0.93), for Think Fast 4.15 (± 0.95), for Numbers 4.12 (± 0.92), for Treasure Hunt 4.20 (± 0.97), for Mixer 4.09 (± 0.9), and for Catch-a-Cloud 3.21 (± 1.24). The ratings increased over time for all games but Catch-a-Cloud, where the rating of the last day decreased: day 1: 2.9 (± 1.35), day 8: 3.42 (± 1.15), and day 11: 3.34 (± 1.12). Mean ratings on the clearness of the instructions were 4.79 (range: 4.67—4.86): Match Maker: 4.67 (± 0.72), Think Fast: 4.82 (± 0.54), Numbers: 4.84 (± 0.5), Treasure Hunt: 4.86 (± 0.41), Mixer: 4.70 (± 0.68), Catch-a-Cloud: 4.84 (± 0.54). Ratings regarding instructions did not change strongly over time. The average rating of the level of frustration was 3.9 (range: 2.83—4.72): Match Maker: 3.6 (± 1.07), Think Fast: 4.18 (± 0.91), Numbers: 4.23 (± 0.87), Treasure Hunt: 2.86 (± 1.29), Mixer: 3.84 (± 1), Catch-a-Cloud: 4.71 (± 0.65). For Match Maker and Treasure Hunt, the frustration rating between day 4 and day 8 decreased by 0.52 and 1.24 points, respectively. The detailed ratings for each day of questionnaire completion are shown in the appendix (Table 18: Questionnaire ratings).

## Discussion

In this study, we investigated the relative difficulty between levels, reliability, practice effects, and ability to measure change in performance over time, as well as the acceptance by participants of CoGames—a smartphone-based application for cognitive games that was developed in-house.

Overall, we were able to quantify the difficulty differences between levels. In most cases, the difficulty increased as intended; however, this was not always the case. All difficulty levels of all games reached our predefined cutoffs for reliability and acceptance (enjoyment). Furthermore, the observed increase in game scores over time suggests the presence of a measurable practice effect. However, the ability to detect change in cognitive function over time has to be further investigated.

### Difficulty level estimation

Using the linear mixed model estimates of every level, we were able to assess the relative difference in performance of each difficulty level compared to the reference (level “Beginner”). These linear mixed models included age, years of education, operating system, and their interactions with the difficulty levels as covariates. A lower level estimate indicated worse performance and consequently higher level difficulty. Overall, most, but not all levels were notably different from the level “Beginner” depending on the game. Especially, lower levels appeared to be similar in difficulty to level “Beginner” (see Table 5, 6, 7, 8, 9, 10, 11 in the appendix). We assume this effect is the result of using the easiest level as the reference. Furthermore, the impact of covariates (age, operating system, and years of education) also varied across games and levels. Interestingly, according to these linear mixed model estimates not all levels increased in difficulty as designed (Level 1 = easiest, Level 8 = most difficult). Of course, these results must be interpreted carefully considering the potential influence of practice effects. In any case, these models quantify the difficulty levels and therefore provide evidence enabling the arrangement of levels toward adaptive games with consistently increasing difficulty. Standardization allows for comparing scores across difficulty levels. This is necessary if we want to monitor performance over time or compare scores cross-sectionally using this difficulty-adjusting system. To this purpose, we propose to standardize the scores according to the following formula:

$$Standardized\; score = \frac{raw\;score - predicted\;score}{{\sqrt {total\;varaince}}}$$

- Raw score: the score achieved in the game (e.g., number of successful responses in 60 s).
- Predicted score: the score based on the linear mixed model depending on the level, age, operating system, and years of education.
- Total variance: variance of the prediction + residual variance.

Importantly, predicted values may vary in different populations.

### Reliability

All difficulty levels across all games reached the predefined cutoff coefficient of |*r*_*s*_* |*≥ 0.6 (range: 0.64—0.94), except for the repetitions of the three easiest difficulty levels (“Beginner”, “Apprentice”, and “Graduate”) of the visual short-term memory game Treasure Hunt. As shown in Table 15, these three lowest levels had a clear ceiling effect with most participants achieving a close to perfect score (median > 90% correct answers). This range restriction results in reduced variability and consequently in a lower correlation coefficient. Supporting this interpretation, the correlation coefficient increased in the higher difficulty levels, in which we observed wider and less skewed score distributions (Table 15).

While the number of studies successfully proving high reliability of self-administered digital versions of established neuropsychological tests (e.g., SDMT) is large [11, 14, 28–32], the published evidence regarding the reliability of gamified smartphone-based cognitive tests is scarce. Nonetheless, the existing literature supports our findings: Brewster et al. (2021): ICC range = 0.85–0.87; Wiley et al. (2024): ICC = 0.58; Staffaroni et al. (2024): ICC range = 0.77–0.95 [33–35]. It is noteworthy that a direct comparison of the referenced studies with the results of CoGames is compromised by the different study designs (frequency, total duration, number of repetitions, etc.) and the timing of test and retest. Nevertheless, all studies suggest that smartphone-based tests can provide a reliable assessment of cognitive performance.

### Change in performance over time

As expected, we found performance improvements in most games when comparing the scores of the easiest level that was repeated at days 1, 2, 6, and 11 [36]. As shown in Fig. 2a–f, the performance of Match Maker, Think Fast, and Mixer showed either a linear increase over time or an initial steeper increase, followed by a plateauing of the curve. No major change was detected in the performance curves for Numbers, Treasure Hunt, and Catch-a-Cloud. As for Treasure Hunt, we attribute this to very low difficulty of the “Beginner” level which led to a ceiling effect. Once this ceiling (i.e., 100% correct responses) is reached, further improvement is impossible. Numbers and Catch-a-Cloud, are reaction time-based and less cognitively demanding games. Such tasks are easier automated and do not require as much practice until a plateau of maximal performance is reached compared to tasks that are more complex and cognitively challenging [37]. Overall, younger age correlated with better scores. However, the practice effect appeared to be similar in all age groups.

In any case, although not definitive proof, the fact by itself that these practice effects were observable suggests that CoGames might have the potential to detect performance changes over time. This is another mandatory prerequisite for a useful monitoring tool, but also an effect that must be considered in clinical monitoring.

### Acceptance by participants: enjoyment

In recent years, awareness of the added value of including gamification elements in digital applications for rehabilitation, therapy, or assessment in MS has gained increasing interest [10, 38, 39]. Factors such as enjoyment, user-friendliness, and meaningfulness are fundamental to achieve high adherence to monitoring tools. In this study, we investigated elements of acceptance on a 5-point Likert scale (1 = worst, and 5 = best), with a focus on enjoyment. Our findings show that all games are at least moderately enjoyed (range: 3.13—4.22). We assume that gamification aspects, such as audible and visual feedback to correct and false responses, colorful design of interactive items and background, and points as rewards for good performance contribute strongly to the enjoyment factor, hence encouraging the acceptance and use of such tools.

In most of our games, the enjoyment rating stayed the same over the three time points (day 4, 8, and 11). Minor differences in enjoyment ratings across the three different rating days might indicate which difficulty levels were preferred: Lower enjoyment ratings during higher levels in the games Treasure Hunt and Match Maker reflect our results, which show that the difficulty increase in those two games was higher compared to the other games. This might have led to more frustration in higher levels of those games. In support of this interpretation, the “frustration” ratings showed similar changes at the same time of questionnaire completion as the enjoyment ratings (Table 18: Questionnaire ratings).

### Acceptance by participants: representation of cognitive function, perceived difficulty increase, clearness of instructions, and frustration

Participants perceived all games as representative of their cognitive abilities and found the difficulty increase well designed, meaning the levels seemed to get more difficult by the day (excluding the repetitions of level 1). The in-app instructions were rated as very clear across all games. Not surprisingly, the frustration ratings correlated strongly with the enjoyment ratings, which is especially visible in the ratings of the higher levels. These findings are comparable to results of our previous study [24].

Overall, the participants found the CoGames enjoyable, representative, and well designed regarding user friendliness, clarity of instructions, and difficulty level transitions, which is an excellent foundation for high adherence when using a monitoring tool.

#### Limitations

We cannot rule out a potential recruitment bias toward participants with a higher affinity for digital devices and gaming. The enjoyment rating might therefore be different in the general population. We attempted to minimize this possible bias by broad recruitment across different age groups not only via digital media (website), but also via flyers in the hospital and public places and offered a small remuneration for participation. The recruitment of healthy volunteers was purposely chosen to optimize reliability testing without disease-related influences. Consequently, the acceptance ratings need to be confirmed in PwMS of different disability levels. Monitoring of cognition using an adaptive difficulty system might also serve as cognitive training which is important to consider when interpreting the data. However, with an adequate frequency we expect the practice effect to be minimal once the individual performance level is reached. Our study population might not have been large enough to be representative of the general population. Therefore, our results need to be replicated in further larger studies with longer follow-up. Lastly, the short study duration did not allow an in-depth and conclusive analysis of practice effects and sensitivity to change.

#### Conclusion and future research

The results of this study support the hypothesis that adaptive, digital, and gamified cognitive tests can be used as a reliable and well-accepted assessment and monitoring tool. Besides improved convenience and enjoyment, CoGames aims to provide a more comprehensive assessment of cognitive function by covering multiple cognitive domains. Whether CoGames can assess specific cognitive domains as intended is further investigated in a currently running validation program, where the adaptive versions of CoGames are being compared both cross-sectionally and longitudinally to established neuropsychological tests in a population of PwMS and healthy volunteers (dreaMS Validation study 1, NCT05009160). Using the thresholds of the 25th and 75th percentile of every level found in the CoGames study, we implemented a dynamic difficulty adjustment (DDA) system, which allows patients to level up and down depending on their individual performance. This adaptive design is intended to promote the state of high concentration, keeping users engaged and motivated by avoiding frustration (task too difficult) and boredom (task too easy). Furthermore, the difficulty level quantification allows us to rearrange the levels for optimal and consistent difficulty increase in the next CoGames version. Lastly, we propose a standardization of the scores, enabling comparisons of performance across different difficulty levels.

## Electronic supplementary material

Below is the link to the electronic supplementary material.

Supplementary file1 (DOCX 13 kb)

Supplementary file2 (MOV 11431 kb)

Supplementary file3 (MOV 40343 kb)

Supplementary file4 (MOV 10032 kb)

Supplementary file5 (MOV 9088 kb)

Supplementary file6 (MOV 12293 kb)

Supplementary file7 (MOV 9706 kb)

## Appendix

See Tables 5, 6, 7, 8, 9, 10, 11, 12, 13, 14, 15, 16, 17, 18.

**Table 5:** Match Maker: Summary of the linear mixed model output

REML criterion at convergence: 6505.3							
Scaled residuals	min	1Q	median	3Q	max		
	−4.9323	−0.5215	0.0334	0.6104	4.4642		

Random effects	variance	std. dev					
user	42.77	6.540					
residual	49.57	7.041					
Number of observations: 932, groups: user, 64							

Fixed effects	estimate	std.error	95% CI lower	95%CI upper	statistic	df	*p*-value
(Intercept)	58.609	5.675	47.29	69.93	10.328	76.69	< 0.001
Apprentice	7.353	3.022	1.431	13.275	2.434	861.852	0.015
Graduate	6.551	2.989	0.672	12.43	2.192	860.078	0.029
Expert	8.089	3.028	2.153	14.025	2.671	860.825	0.008
Star	−12.515	2.953	−18.293	−6.737	−4.237	860.894	< 0.001
Superstar	−7.686	2.994	−13.556	−1.816	−2.567	861.283	0.01
Hero	−6.36	3.075	−12.391	−0.329	−2.068	862.519	0.039
Deity	−16.468	3.071	−22.489	−10.447	−5.363	861.905	< 0.001
Age	−0.445	0.075	−0.594	−0.296	−5.939	127.176	< 0.001
OS(iOS)	16.669	2.132	12.454	20.884	7.82	131.702	< 0.001
Years of education	0.646	0.337	−0.027	1.319	1.917	59.644	0.06
Apprentice: Age	0.009	0.066	−0.119	0.137	0.133	862.479	0.894
Graduate: Age	0.069	0.064	−0.056	0.194	1.07	859.627	0.285
Expert: Age	0.02	0.065	−0.107	0.147	0.314	860.32	0.754
Star: Age	0.006	0.064	−0.120	0.132	0.094	861.271	0.925
Superstar: Age	−0.028	0.065	−0.156	0.1	−0.432	861.315	0.666
Hero: Age	0	0.067	−0.131	0.131	−0.004	862.991	0.997
Deity: Age	0.09	0.066	−0.040	0.22	1.364	862.161	0.173
Apprentice: OS(iOS)	1.427	1.869	−2.237	5.091	0.763	860.747	0.445
Graduate: OS(iOS)	2.011	1.893	−1.702	5.724	1.063	860.441	0.288
Expert: OS(iOS)	−0.192	1.898	−3.913	3.529	−0.101	860.744	0.92
Star: OS(iOS)	−13.564	1.842	−17.175	−9.953	−7.362	860.235	< 0.001
Superstar: OS(iOS)	−9.528	1.867	−13.188	−5.868	−5.104	860.456	< 0.001
Hero: OS(iOS)	−9.122	1.877	−12.801	−5.443	−4.86	861.055	< 0.001
Deity: OS(iOS)	−13.944	1.901	−17.670	−10.218	−7.336	861.097	< 0.001

**Table 6:** Think fast: summary of the linear mixed model output

REML criterion at convergence: 7615.3							
Scaled residuals	Min	1Q	Median	3Q	Max		
	−3.4967	−0.6389	0.0162	0.6403	2.6171		

Random effects	Variance	Std. Dev					
User	131.3	11.46					
Residual	159.2	12.62					
Number of observations: 949, groups: user, 64							

Fixed effects	Estimate	Std.error	95% CI lower	95%CI upper	Statistic	df	*p*-value
(Intercept)	125.864	9.955	105.982	145.746	12.643	75.756	< 0.001
Apprentice	−1.536	5.372	−12.078	9.006	−0.286	865.967	0.775
Graduate	−12.672	5.311	−23.086	−2.258	−2.386	864.306	0.017
Expert	−12.768	5.391	−23.335	−2.201	−2.368	865.111	0.018
Star	−12.004	5.248	−22.307	−1.701	−2.287	865.122	0.022
Superstar	−28.616	5.301	−38.995	−18.237	−5.398	865.457	< 0.001
Hero	−36.505	5.411	−47.127	−25.883	−6.746	865.994	< 0.001
Deity	−26.87	5.467	−37.596	−16.144	−4.915	866.381	< 0.001
Age	−1.247	0.131	−1.506	−0.988	−9.489	125.739	< 0.001
OS (iOS)	5.533	3.739	−1.871	12.937	1.48	130.259	0.141
Years of education	0.447	0.591	−0.734	1.628	0.757	58.885	0.452
Apprentice: age	0.233	0.118	0.001	0.465	1.977	866.573	0.048
Graduate: age	0.503	0.114	0.279	0.727	4.422	863.902	< 0.001
Expert: age	0.376	0.116	0.149	0.603	3.25	864.654	0.001
Star: age	0.445	0.115	0.219	0.671	3.884	865.557	< 0.001
Superstar: age	0.573	0.115	0.347	0.799	4.998	865.616	< 0.001
Hero: age	0.703	0.119	0.469	0.937	5.915	866.558	< 0.001
Deity: age	0.605	0.117	0.375	0.835	5.164	866.403	< 0.001
Apprentice: OS (iOS)	−1.736	3.323	−8.256	4.784	−0.522	864.908	0.602
Graduate: OS (iOS)	−3.262	3.362	−9.858	3.334	−0.97	864.629	0.332
Expert: OS (iOS)	−1.274	3.372	−7.874	5.326	−0.378	864.999	0.706
Star: OS (iOS)	−7.255	3.283	−13.692	−0.818	−2.21	864.497	0.027
Superstar: OS (iOS)	−4.481	3.292	−10.937	1.975	−1.361	864.659	0.174
Hero: OS (iOS)	−5.528	3.325	−12.055	1.001	−1.662	864.871	0.097
Deity: OS (iOS)	−4.65	3.382	−11.283	1.983	−1.375	865.448	0.17

**Table 7:** Numbers: summary of the linear mixed model output

REML criterion at convergence: 5857.2							
Scaled residuals	Min	1Q	Median	3Q	Max		
	−4.9823	−0.5702	0.0375	0.6640	2.8376		

Random effects	Variance	Std. Dev					
User	34.81	5.9					
Residual	23.04	4.8					
Number of observations: 948, groups: user, 64							

Fixed effects	Estimate	Std.error		95% CI lower			95%CI upper	Statistic		df			*p*-value
(Intercept)	50.882	4.912		41.085			60.679	10.36		68.287			< 0.001
Apprentice	−0.963	2.046		−4.975			3.049	−0.471		863.788			0.638
Graduate	2.572	2.023		−1.391			6.535	1.271		862.699			0.204
Expert	−3.936	2.05		−7.955			0.083	−1.921		863.117			0.055
Star	−7.121	1.999		−11.041			−3.201	−3.562		863.242			< 0.001
Superstar	−6.579	2.028		−10.555			−2.603	−3.245		863.463			0.001
Hero	−6.274	2.083		−10.359			−2.189	−3.012		864.175			0.003
Deity	−8.146	2.089		−12.245			−4.047	−3.899		864.062			< 0.001
Age	−0.235	0.062		−0.358			−0.112	−3.801		94.272			< 0.001
OS (iOS)	−0.621	1.752		−4.085			2.843	−0.354		96.453			0.724
Years of education	0.863	0.299		0.265			1.461	2.884		58.896			0.005
Apprentice: age	−0.026	0.045		−0.114			0.062	−0.578		864.18			0.563
Graduate: age	−0.102	0.043		−0.186			−0.018	−2.358		862.46			0.019
Expert: age	0.001	0.044		−0.086			0.088	0.015		862.85			0.988
Star: age	−0.008	0.044		−0.095			0.079	−0.174		863.497			0.862
Superstar: age	−0.056	0.044		−0.142			0.03	−1.289		863.561			0.198
Hero: age	−0.084	0.046		−0.174			0.006	−1.842		864.471			0.066
Deity: age	−0.113	0.045		−0.202			−0.024	−2.528		864.081			0.012
Apprentice: OS (iOS)	−1.908	1.264		−4.387			0.571	−1.509		863.106			0.132
Graduate: OS (iOS)	−2.945	1.279		−5.454			−0.436	−2.303		862.878			0.022
Expert: OS (iOS)	−2.165	1.282		−4.679			0.349	−1.689		863.053			0.092
Star: OS (iOS)	−1.852	1.246		−4.294			0.59	−1.487		862.809			0.137
Superstar: OS (iOS)	−0.647	1.26		−3.119			1.825	−0.514		862.98			0.608
Hero:OS(iOS)	−1.015	1.27		−3.507			1.477	−0.8		863.288			0.424
Deity: OS (iOS)	−0.158	1.292		−2.692			2.376	−0.123		863.457			0.902

**Table 8:** Treasure hunt: summary of the linear mixed model output

REML criterion at convergence: 7420.3							
Scaled residuals	Min	1Q	Median	3Q	Max		
	−5.3100	−0.5720	0.0914	0.6324	2.5461		

Random effects	variance	std. dev					
User	114.3	10.69					
Residual	149.9	12.24					
Number of observations: 932, groups: user, 64							

Fixed effects		Estimate	Std.error		95% CI lower	95%CI upper			Statistic	df			*p*-value
(Intercept)		87.825	9.384		69.159	106.491			9.359	77.811			< 0.001
Apprentice		−7.156	5.206		−17.366	3.054			−1.375	849.404			0.17
Graduate		−9.913	5.181		−20.085	0.259			−1.913	847.69			0.056
Expert		−14.478	5.248		−24.778	−4.178			−2.759	848.477			0.006
Star		−16.319	5.112		−26.349	−6.289			−3.192	848.469			0.001
Superstar		−9.472	5.19		−19.645	0.701			−1.825	848.899			0.068
Hero		−15.93	5.326		−26.391	−5.469			−2.991	850.174			0.003
Deity		−19.106	5.323		−29.541	−8.671			−3.589	849.941			< 0.001
Age		−0.159	0.125		−0.406	0.088			−1.274	132.568			0.205
OS (iOS)		1.687	3.551		−5.328	8.702			0.475	137.4			0.636
Years of education		0.887	0.554		−0.22	1.994			1.6	59.443			0.115
Apprentice: age		0.079	0.114		−0.145	0.303			0.689	850.04			0.491
Graduate: age		−0.015	0.111		−0.233	0.203			−0.139	847.276			0.889
Expert: age		−0.026	0.113		−0.247	0.195			−0.228	847.984			0.82
Star: age		−0.138	0.111		−0.356	0.08			−1.242	848.865			0.215
Superstar: age		−0.415	0.112		−0.635	−0.195			−3.699	849.007			< 0.001
Hero: age		−0.261	0.117		−0.49	−0.032			−2.235	850.649			0.026
Deity: age		−0.171	0.114		−0.394	0.052			−1.496	849.94			0.135
Apprentice: OS (iOS)		1.739	3.232		−4.598	8.076			0.538	848.312			0.591
Graduate: OS (iOS)		4.321	3.283		−2.114	10.756			1.316	848.045			0.188
Expert: OS (iOS)		4.976	3.288		−1.482	11.434			1.513	848.469			0.131
Star: OS (iOS)		0.615	3.189		−5.637	6.867			0.193	847.794			0.847
Superstar: OS (iOS)		1.88	3.225		−4.45	8.21			0.583	848.125			0.56
Hero: OS (iOS)		−1.107	3.25		−7.482	5.268			−0.341	848.676			0.733
Deity: OS (iOS)		0.415	3.294		−6.041	6.871			0.126	848.972			0.9

**Table 9:** Mixer: summary of the linear mixed model output

REML criterion at convergence: 6182.2							
Scaled residuals	Min	1Q	Median	3q	Max		
	−4.7120	−0.5722	0.0874	0.6182	3.0594		

Random effects	Variance	Std. dev					
User	34.67	6.609					
Residual	33.28	5.769					
Number of observations: 947, groups: user, 64							

Fixed effects	Estimate	Std.error	95% CI lower	95%CI upper	Statistic	df	*p*-value
(Intercept)	50.485	5.547	39.426	61.544	9.102	70.222	< 0.001
Apprentice	−8.381	2.459	−13.212	−3.55	−3.408	863.503	0.001
Graduate	−2.656	2.432	−7.426	2.114	−1.092	862.268	0.275
Expert	−9.674	2.464	−14.507	−4.841	−3.927	862.752	< 0.001
Star	−9.589	2.406	−14.309	−4.869	−3.986	862.892	< 0.001
Superstar	−4.91	2.436	−9.693	−0.127	−2.015	863.13	0.044
Hero	−10.389	2.503	−15.296	−5.482	−4.15	863.942	< 0.001
Deity	−7.545	2.503	−12.452	−2.638	−3.015	863.782	0.003
Age	−0.298	0.071	−0.439	−0.157	−4.216	101.376	< 0.001
OS (iOS)	1.059	2.003	−2.91	5.028	0.529	104.084	0.598
Years of education	0.714	0.336	0.042	1.386	2.126	59.352	0.038
Apprentice: age	0.065	0.054	−0.041	0.171	1.203	863.999	0.229
Graduate: age	0.071	0.052	−0.031	0.173	1.356	862.045	0.175
Expert: age	0.077	0.053	−0.027	0.181	1.458	862.491	0.145
Star: age	0.1	0.053	−0.003	0.203	1.905	863.226	0.057
Superstar: age	0.048	0.053	−0.056	0.152	0.919	863.28	0.358
Hero: age	0.076	0.055	−0.032	0.184	1.384	864.335	0.167
Deity: age	−0.025	0.054	−0.131	0.081	−0.472	863.87	0.637
Apprentice: OS (iOS)	−3.178	1.525	−6.168	−0.188	−2.084	862.79	0.037
Graduate: OS (iOS)	−3.025	1.544	−6.057	0.007	−1.959	862.525	0.05
Expert: OS (iOS)	−2.428	1.546	−5.459	0.603	−1.57	862.733	0.117
Star: OS (iOS)	−4.082	1.505	−7.033	−1.131	−2.713	862.463	0.007
Superstar: OS (iOS)	−4.752	1.517	−7.728	−1.776	−3.131	862.635	0.002
Hero: OS (iOS)	−0.589	1.531	−3.591	2.413	−0.384	862.999	0.701
Deity: OS (iOS)	−3.014	1.552	−6.058	0.03	−1.942	863.168	0.053

**Table 10:** Catch-a-cloud test mode: summary of the linear mixed model output

REML criterion at convergence: 4581							
Scaled residuals	Min	1Q	Median	3Q	Max		
	−5.3095	−0.4866	0.0463	0.5962	3.1591		

Random effects	Variance	Std. dev					
User	11.745	3.427					
Residual	5.859	2.421					
Number of observations: 943, groups: user, 64							

Fixed effects	Estimate		Std.error		95% CI lower		95%CI upper	Statistic	df			*p*-value
(Intercept)	35.576		2.816		29.955		41.197	12.631	67.214			< 0.001
Apprentice	1.37		1.035		−0.662		3.402	1.325	865.361			0.186
Graduate	0.376		1.028		−1.64		2.392	0.366	864.447			0.715
Expert	1.234		1.04		−0.807		3.275	1.186	864.78			0.236
Star	0.484		1.015		−1.507		2.475	0.476	864.943			0.634
Superstar	0.933		0.847		−0.728		2.594	1.102	865.64			0.271
Age	v0.243		0.035		−0.313		−0.173	−6.962	87.07			< 0.001
OS (iOS)	5.13		0.99		3.161		7.099	5.184	89.523			< 0.001
Years of education	0.12		0.173		−0.226		0.466	0.695	59.79			0.49
Apprentice: age	0.018		0.023		−0.027		0.063	0.788	865.664			0.431
Graduate: age	0.052		0.022		0.008		0.096	2.346	864.307			0.019
Expert: age	0.049		0.022		0.005		0.093	2.19	864.613			0.029
Star:Age	0.043		0.022		−0.0001		0.087	1.967	865.133			0.05
Superstar: age	0.046		0.018		0.01		0.082	2.532	865.918			0.012
Apprentice: OS (iOS)	0.349		0.643		−0.913		1.611	0.542	864.828			0.588
Graduate: OS (iOS)	1.67		0.652		0.392		2.948	2.561	864.541			0.011
Expert: OS (iOS)	1.049		0.652		−0.23		2.328	1.608	864.673			0.108
Star: OS (iOS)	2.509		0.635		1.264		3.754	3.952	864.61			< 0.001
Superstar: OS (iOS)	1.698		0.528		0.662		2.734	3.218	865.056			0.001

**Table 11:** Catch-a-cloud game mode: summary of the linear mixed model output

REML criterion at convergence: 5260.9							
Scaled residuals	Min	1Q	Median	3Q	Max		
	−6.3295	−0.5199	0.0121	0.5488	5.8869		

Random effects	Variance	Std. dev					
User	23.72	4.870					
Residual	12.26	3.501					
Number of observations: 943, groups: user, 64							

Fixed effects	Estimate	Std.error	95% CI lower	95%CI upper	Statistic	df	*p*-value
(Intercept)	54.123	4.008	46.111	67.336	13.502	67.336	< 0.001
Apprentice	−1.294	1.496	−4.268	1.681	−0.865	865.285	0.388
Graduate	1.487	1.487	−1.438	4.412	1	864.34	0.318
Expert	−2.613	1.505	−5.576	0.35	−1.737	864.685	0.083
Star	0.591	1.469	−2.295	2.477	0.403	864.852	0.687
Superstar	4.194	1.225	1.863	6.524	3.424	865.573	0.001
Age	−0.33	0.05	−0.431	−0.23	−6.637	87.915	< 0.001
OS (iOS)	7.702	1.412	5.456	9.948	5.456	90.462	< 0.001
Years of education	0.265	0.246	−0.219	0.749	1.077	59.661	0.286
Apprentice: age	0.043	0.033	−0.021	0.107	1.303	865.598	0.193
Graduate: age	0.057	0.032	−0.007	0.121	1.785	864.196	0.075
Expert: age	0.113	0.032	0.05	0.175	3.51	864.512	< 0.001
Star: age	0.084	0.032	0.021	0.148	2.643	865.048	0.008
Superstar: age	0.068	0.026	0.017	0.119	2.571	865.859	0.01
Apprentice: OS (iOS)	1.821	0.93	−0.014	3.656	1.957	864.735	0.051
Graduate: OS (iOS)	2.212	0.943	0.365	4.059	2.346	864.439	0.019
Expert: OS (iOS)	0.909	0.944	−0.944	1.963	0.964	864.575	0.335
Star: OS (iOS)	2.081	0.918	0.298	3.864	2.266	864.508	0.024
Superstar: OS (iOS)	0.75	0.763	−0.747	2.247	0.982	864.969	0.326

**Table 12:** Match Maker: Achieved scores and rho (test–retest reliability) by study day and difficulty level

Day	Level	n	Min	Q1	Median	Mean	Q3	Max	rho	*p*-value
Day 1	Beginner	72	28	51.75	62	61.51	72.00	94	0.79	< 0.001
Day 2	Beginner	70	25	61.00	68	69.45	79.00	101	0.93	< 0.001
Day 3	Apprentice	69	27	61.00	69	68.40	79.75	97	0.88	< 0.001
Day 4	Graduate	68	35	65.00	71	71.81	80.25	100	0.93	< 0.001
Day 5	Expert	66	27	61.00	68	69.47	80.00	96	0.91	< 0.001
Day 6	Beginner	67	29	67.00	74	75.25	86.00	99	0.90	< 0.001
Day 7	Star	73	19	31.00	40	40.00	47.75	80	0.73	< 0.001
Day 8	Superstar	72	21	38.00	46	46.74	55.50	72	0.77	< 0.001
Day 9	Hero	66	24	42.75	49	48.86	57.25	76	0.83	< 0.001
Day 10	Deity	66	20	33.00	39	39.36	45.25	61	0.77	< 0.001
Day 11	Beginner	69	33	64.00	72	72.63	81.00	101	0.89	< 0.001

**Table 13:** Think Fast: Achieved scores and rho (test–retest reliability) by study day and difficulty level

Day	Level	n	Min	Q1	Median	Mean	Q3	Max	rho	*p*-value
Day 1	Beginner	76	15	67.75	87.0	86.29	105.25	138	0.92	< 0.001
Day 2	Beginner	71	49	80.00	103.0	101.40	122.75	157	0.92	< 0.001
Day 3	Apprentice	69	45	77.00	89.5	91.20	108.00	138	0.86	< 0.001
Day 4	Graduate	68	51	79.00	89.0	91.26	104.00	134	0.82	< 0.001
Day 5	Expert	67	37	69.25	83.0	86.46	103.75	138	0.83	< 0.001
Day 6	Beginner	69	58	90.00	112.5	112.11	132.00	171	0.86	< 0.001
Day 7	Star	73	38	74.00	87.0	87.14	99.75	144	0.81	< 0.001
Day 8	Superstar	74	26	64.00	77.0	77.53	93.00	133	0.74	< 0.001
Day 9	Hero	66	34	61.00	74.5	75.99	87.25	132	0.79	< 0.001
Day 10	Deity	66	35	65.00	81.5	81.34	95.50	139	0.80	< 0.001
Day 11	Beginner	68	61	98.00	116.0	119.30	141.25	189	0.91	< 0.001

**Table 14:** Numbers: achieved scores and rho (test–retest reliability) by study day and difficulty level

Day	Level	n	min	Q1	Median	Mean	Q3	Max	rho	*p*-value
Day 1	Beginner	75	38	49.25	54.0	54.02	58.00	72	0.82	< 0.001
Day 2	Beginner	69	40	51.00	55.5	55.06	59.75	72	0.81	< 0.001
Day 3	Apprentice	69	24	46.00	51.0	51.00	57.00	72	0.81	< 0.001
Day 4	Graduate	69	30	45.25	50.0	50.60	57.75	74	0.85	< 0.001
Day 5	Expert	67	20	43.00	48.0	48.52	54.00	72	0.67	< 0.001
Day 6	Beginner	68	37	51.75	56.0	55.62	60.00	72	0.72	< 0.001
Day 7	Star	73	13	39.00	45.0	45.21	52.00	68	0.78	< 0.001
Day 8	Superstar	72	13	38.75	44.0	44.75	50.25	66	0.78	< 0.001
Day 9	Hero	67	20	38.00	44.0	44.08	50.00	61	0.66	< 0.001
Day 10	Deity	66	10	35.00	41.0	40.88	48.00	62	0.75	< 0.001
Day 11	Beginner	69	33	52.00	56.0	55.58	60.00	76	0.81	< 0.001

**Table 15:** Treasure Hunt: achieved scores and rho (test–retest reliability) by study day and difficulty level

Day	Level	n	Min	Q1	Median	Mean	Q3	Max	rho	*p*-value
Day 1	Beginner	74	71.4	95.24	100.00	95.83	100.00	100	0.48	< 0.001
Day 2	Beginner	68	76.6	95.83	100.00	97.19	100.00	100	0.21	0.093
Day 3	Apprentice	68	66.1	88.57	94.44	93.19	100.00	100	0.38	0.001
Day 4	Graduate	65	48.6	81.09	91.67	88.42	97.44	100	0.55	< 0.001
Day 5	Expert	64	43.5	74.11	85.42	83.70	95.89	100	0.67	< 0.001
Day 6	Beginner	67	68.8	100.00	100.00	98.02	100.00	100	0.32	0.009
Day 7	Star	71	22.2	63.44	75.87	74.01	87.04	100	0.64	< 0.001
Day 8	Superstar	70	11.1	53.70	70.37	70.63	90.05	100	0.74	< 0.001
Day 9	Hero	65	0.0	53.01	72.69	69.64	89.58	100	0.73	< 0.001
Day 10	Deity	65	3.7	56.77	73.78	70.87	91.67	100	0.72	< 0.001
Day 11	Beginner	66	75.0	100.00	100.00	98.76	100.00	100	0.21	0.087

**Table 16:** Mixer: achieved scores and rho (test–retest reliability) by study day and difficulty level

Day	Level	n	Min	Q1	Median	Mean	Q3	Max	rho	*p*-value
Day 1	Beginner	74	20	45.00	51.0	50.74	56.00	73	0.77	< 0.001
Day 2	Beginner	70	36	50.00	56.5	56.34	62.25	79	0.88	< 0.001
Day 3	Apprentice	69	14	35.00	43.0	43.06	52.00	66	0.84	< 0.001
Day 4	Graduate	68	26	43.75	49.0	48.88	55.00	70	0.81	< 0.001
Day 5	Expert	67	19	37.00	42.5	42.37	48.00	67	0.82	< 0.001
Day 6	Beginner	65	30	52.00	58.5	58.60	65.00	80	0.87	< 0.001
Day 7	Star	72	18	35.00	42.0	41.92	49.25	66	0.78	< 0.001
Day 8	Superstar	73	12	37.25	45.0	44.76	52.00	75	0.76	< 0.001
Day 9	Hero	67	12	38.00	43.0	42.60	50.00	63	0.79	< 0.001
Day 10	Deity	67	14	33.00	40.0	40.28	49.00	60	0.82	< 0.001
Day 11	Beginner	66	36	54.00	59.0	60.13	65.00	83	0.92	< 0.001

**Table 17:** Catch-a-cloud (game mode): achieved scores and rho (test–retest reliability) by study day and difficulty level

Day	Level	n	Min	Q1	Median	Mean	Q3	Max	rho	*p*-value
Day 1	Beginner	72	31	45	50.0	50.28	55.00	67	0.90	< 0.001
Day 2	Beginner	69	23	46	50.5	51.07	57.00	80	0.94	< 0.001
Day 3	Apprentice	70	31	45	49.5	50.59	57.00	68	0.92	< 0.001
Day 4	Graduate	68	38	49	55.0	55.43	62.00	75	0.93	< 0.001
Day 5	Expert	67	31	47	52.0	52.44	58.00	70	0.89	< 0.001
Day 6	Beginner	66	36	48	52.5	53.01	58.00	71	0.86	< 0.001
Day 7	Star	73	34	49	55.5	55.02	61.00	74	0.86	< 0.001
Day 8	Superstar	70	39	53	57.0	56.75	62.00	72	0.84	< 0.001
Day 9	Hero	67	38	52	58.0	57.58	62.75	75	0.86	< 0.001
Day 10	Deity	66	40	54	58.0	57.68	63.00	77	0.90	< 0.001
Day 11	Beginner	69	31	47	54.0	53.40	60.00	71	0.94	< 0.001

**Table 18:** Questionnaire results (mean rating ± SD) on a Likert scale from 0–5

Enjoyment				
Game	Day 4	Day 8	Day 11	Mean
Match maker	4.04 (± 0.92)	3.60 (± 0.92)	3.73 (± 0.98)	3.8 (± 0.96)
Think fast	4.25 (± 0.75)	4.22 (± 0.5)	4.39 (± 0.74)	4.28 (± 0.74)
Numbers	4.13 (± 0.93)	4.19 (± 0.76)	4.09 (± 0.87)	4.14 (± 0.85)
Treasure hunt	3.47 (± 1.15)	2.78 (± 1.23)	3.13 (± 1.1)	3.13 (± 1.19)
Mixer	3.66 (± 1.12)	3.75 (± 1)	3.81 (± 0.91)	3.74 (± 1.01)
Catch-a-Cloud	4.10 (± 1.06)	4.30 (± 0.98)	4.27 (± 0.96)	4.22 (± 1)

Representation of cognitive function				
Game	Day 4	Day 8	Day 11	Mean
Match maker	3.86 (± 1)	3.82 (± 0.86)	3.97 (± 0.89)	3.88 (± 0.92)
Think fast	3.74 (± 0.86)	3.90 (± 0.92)	4.18 (± 0.6)	3.94 (± 0.83)
Numbers	3.74 (± 0.94)	3.82 (± 0.99)	3.94 (± 0.81)	3.83 (± 0.92)
Treasure hunt	3.73 (± 1.03)	3.37 (± 1.21)	3.72 (± 1.19)	3.6 (± 1.15)
Mixer	3.83 (± 1.02)	3.89 (± 0.98)	3.90 (± 0.91)	3.87 (± 0.97)
Catch-a-cloud	3.17 (± 1.3)	3.38 (± 1.32)	3.22 (± 1.29)	3.26 (± 1.3)

Perceived difficulty increase				
Game	Day 4	Day 8	Day 11	Mean
Match maker	3.83 (± 1.09)	4.42 (± 0.74)	4.48 (± 0.75)	4.23 (± 0.93)
Think fast	3.57 (± 1.15)	4.41 (± 0.66)	4.48 (± 0.61)	4.15 (± 0.95)
Numbers	3.94 (± 1.04)	4.16 (± 0.83)	4.30 (± 0.82)	4.12 (± 0.92)
Treasure hunt	3.83 (± 0.98)	4.27 (± 0.98)	4.51 (± 0.82)	4.20 (± 0.97)
Mixer	3.86 (± 1.05)	4.11 (± 0.83)	4.33 (± 0.7)	4.09 (± 0.9)
Catch-a-cloud	2.9 (± 1.35)	3.42 (± 1.15)	3.34 (± 1.12)	3.21 (± 1.24)

Clarity of instructions				
Game	Day 4	Day 8	Day 11	Mean
Match maker	4.69 (± 0.61)	4.73 (± 0.61)	4.60 (± 0.92)	4.67 (± 0.72)
Think fast	4.77 (± 0.6)	4.81(± 0.59)	4.88 (± 0.37)	4.82 (± 0.54)
Numbers	4.78 (± 0.62)	4.86 (± 0.48)	4.90 (± 0.35)	4.84 (± 0.5)
Treasure hunt	4.86 (± 0.35)	4.85 (± 0.49)	4.88 (± 0.37)	4.86 (± 0.41)
Mixer	4.61 (± 0.81)	4.75 (± 0.6)	4.73 (± 0.59)	4.70 (± 0.68)
Catch-a-cloud	4.84 (± 0.51)	4.84 (± 0.58)	4.85 (± 0.53)	4.84 (± 0.54)

Frustration				
Game	Day 4	Day 8	Day 11	Mean
Match maker	4.01 (± 0.99)	3.49 (± 1)	3.30 (± 1.1)	3.6 (± 1.07)
Think fast	4.14 (± 1.02)	4.22 (± 0.85)	4.18 (± 0.83)	4.18 (± 0.91)
Numbers	4.16 (± 0.9)	4.27 (± 0.8)	4.25 (± 0.91)	4.23 (± 0.87)
Treasure hunt	3.62 (± 1.17)	2.38 (± 1.14)	2.49 (± 1.17)	2.86 (± 1.29)
Mixer	3.79 (± 1.1)	3.78 (± 0.98)	3.96 (± 0.91)	3.84 (± 1)
Catch-a-cloud	4.64 (± 0.76)	4.67 (± 0.69)	4.85 (± 0.44)	4.71 (± 0.65)

## References

[1] Lassmann H (2018) Multiple sclerosis pathology. Cold Spring Harb Perspect Med 8(3):1–16. 10.1101/cshperspect.a028936 10.1101/cshperspect.a028936 PMC5830904 29358320

[2] Walton C, King R, Rechtman L et al. (2020) Rising prevalence of multiple sclerosis worldwide: Insights from the Atlas of MS, third edition. Multiple Sclerosis Journal 26(14):1816–1821, 10.1177/1352458520970841 10.1177/1352458520970841 PMC7720355 33174475

[3] Benedict RHB, Fischer JS, Archibald CJ et al (2002) Minimal neuropsychological assessment of MS patients: A consensus approach. Clinical Neuropsychologist 16(3):381–397. 10.1076/clin.16.3.381.13859 12607150 10.1076/clin.16.3.381.13859

[4] Opara JA, Jaracz K, Brola W (2010) Quality of life in multiple sclerosis. J Med Life 3(4):352–358. 10.15844/pedneurbriefs-34-14 21254730 PMC3019078

[5] Strober LB, Rao SM, Lee JC, Fischer E, Rudick R (2014) Cognitive impairment in multiple sclerosis: An 18 year follow-up study. Mult Scler Relat Disord 3(4):473–481. 10.1016/j.msard.2014.03.004 25877059 10.1016/j.msard.2014.03.004

[6] Messinis L, Kosmidis MH, Lyros E, Papathanasopoulos P (2010) Assessment and rehabilitation of cognitive impairment in multiple sclerosis. Int Rev Psychiatry 22(1):22–34. 10.3109/09540261003589372 20233112 10.3109/09540261003589372

[7] De Angelis M, Lavorgna L, Carotenuto A et al (2021) Digital technology in clinical trials for multiple sclerosis: systematic review. J Clin Med 10(11):2328. 10.3390/jcm10112328 34073464 10.3390/jcm10112328 PMC8199078

[8] Gromisch ES, Turner AP, Haselkorn JK, Lo AC, Agresta T (2021) Mobile health (mHealth) usage, barriers, and technological considerations in persons with multiple sclerosis: A literature review. JAMIA Open 4(3):1–10. 10.1093/jamiaopen/ooaa067 10.1093/jamiaopen/ooaa067 PMC8423420 34514349

[9] Bonnechère B, Van Vooren M, Bier JC et al (2018) the use of mobile games to assess cognitive function of elderly with and without cognitive impairment. J Alzheimers Dis 64(4):1285–1293. 10.3233/JAD-180224 29991133 10.3233/JAD-180224

[10] Hsu WY, Rowles W, Anguera J et al. (2021) Application of an adaptive, digital, game-based approach for cognitive assessment in multiple sclerosis: observational study. J Med Internet Res 10.2196/24356 10.2196/24356 PMC7840186 33470940

[11] Maillart E, Labauge P, Cohen M et al (2020) MSCopilot, a new multiple sclerosis self-assessment digital solution: results of a comparative study versus standard tests. Eur J Neurol 27(3):429–436. 10.1111/ene.14091 31538396 10.1111/ene.14091

[12] Rubin LH, Severson J, Marcotte TD et al. (2021) Tablet-based cognitive impairment screening for adults with hiv seeking clinical care: Observational study. JMIR Ment Health 10.2196/25660 10.2196/25660 PMC8461534 34499048

[13] Papp KV, Samaroo A, Chou H et al (2021) Unsupervised mobile cognitive testing for use in preclinical Alzheimer’s disease. Alzheimer’s & Dementia: Diagnosis, Assessment & Disease Monitoring 13(1):1–10. 10.1002/dad2.12243 10.1002/dad2.12243 PMC8481881 34621977

[14] Lam KH, van Oirschot P, den Teuling B et al. (2021) Reliability, construct and concurrent validity of a smartphone-based cognition test in multiple sclerosis. Multiple Sclerosis Journal, 10.1177/13524585211018103 10.1177/13524585211018103 PMC8795217 34037472

[15] Jung HT, Daneault JF, Lee H et al (2019) Remote assessment of cognitive impairment level based on serious mobile game performance: an initial proof of concept. IEEE J Biomed Health Inform 23(3):1269–1277. 10.1109/JBHI.2019.2893897 30668485 10.1109/JBHI.2019.2893897

[16] Petrova-Antonova D, Spasov I, Petkova Y, Manova I, Ilieva S (2020) Cognisoft: A platform for the automation of cognitive assessment and rehabilitation of multiple sclerosis. Computers 9(4):1–13. 10.3390/computers9040093

[17] Dillenseger A, Weidemann ML, Trentzsch K et al (2021) Digital biomarkers in multiple sclerosis. Brain Sci 11(11):1–26. 10.3390/brainsci11111519 10.3390/brainsci11111519 PMC8615428 34827518

[18] Cerrato A, Ponticorvo M (2017) Enhancing Neuropsychological Testing with Gamification and Tangible Interfaces: The Baking Tray Task. Springer International Publishing Vol 10338. 10.1007/978-3-319-59773-7

[19] Skalski P, Dalisay F, Kushin M, Liu Y (2006) Need for Presence and Other Motivations for Video Game Play across Genres. AcademiaEdu.

[20] Cheng VWS, Davenport T, Johnson D, Vella K, Hickie IB (2019) Gamification in apps and technologies for improving mental health and well-being: systematic review. JMIR Ment Health 6(6):e13717. 10.2196/13717 31244479 10.2196/13717 PMC6617915

[21] Vorderer P, Hartmann T, Klimmt C (2003) Explaining the enjoyment of playing video games: the role of competition, 10.1145/958720.958735

[22] Khoshnoud S, Igarzábal FA, Wittmann M (2020) Peripheral-physiological and neural correlates of the flow experience while playing video games: A comprehensive review. PeerJ, 10.7717/peerj.10520 10.7717/peerj.10520 PMC7751419 33384898

[23] Michailidis L, Balaguer-Ballester E, He X (2018) Flow and immersion in video games: The aftermath of a conceptual challenge. Front Psychol 10.3389/fpsyg.2018.01682 10.3389/fpsyg.2018.01682 PMC6134042 30233477

[24] Pless S, Woelfle T, Naegelin Y et al (2023) Assessment of cognitive performance in multiple sclerosis using smartphone-based training games: a feasibility study. J Neurol 270(7):3451–3463. 10.1007/s00415-023-11671-9 36952010 10.1007/s00415-023-11671-9 PMC10267276

[25] RC2NB - dreaMS. https://rc2nb.unibas.ch/en/research/#c520

[26] INDIVI AG. https://www.indivi.health/

[27] Bergendal G, Fredrikson S, Almkvist O (2007) Selective decline in information processing in subgroups of multiple sclerosis: An 8-year longitudinal study. Eur Neurol 57(4):193–202. 10.1159/000099158 17272938 10.1159/000099158

[28] Pham L, Harris T, Varosanec M, Morgan V, Kosa P, Bielekova B (2021) Smartphone-based symbol-digit modalities test reliably captures brain damage in multiple sclerosis. NPJ Digit Med 4(1):1–13. 10.1038/s41746-021-00401-y 33627777 10.1038/s41746-021-00401-y PMC7904910

[29] Woelfle T, Pless S, Reyes O et al (2022) Reliability and acceptance of dreaMS, a software application for people with multiple sclerosis – a feasibility study. J Neurol 270:262–271. 10.1007/s00415-022-11306-5 36042020 10.1007/s00415-022-11306-5 PMC9427170

[30] Patel VP, Shen L, Rose J, Feinstein A (2019) Taking the tester out of the SDMT: A proof of concept fully automated approach to assessing processing speed in people with MS. Mult Scler J 25(11):1506–1513. 10.1177/1352458518792772 10.1177/1352458518792772 30079822

[31] Montalban X, Graves J, Midaglia L et al. (2021) A smartphone sensor-based digital outcome assessment of multiple sclerosis. Multiple Sclerosis Journal, 10.1177/13524585211028561 10.1177/13524585211028561 PMC8961252 34259588

[32] van Oirschot P, Heerings M, Wendrich K, den Teuling B, Martens MB, Jongen PJ (2020) Symbol digit modalities test variant in a smartphone app for persons with multiple sclerosis: validation study. JMIR Mhealth Uhealth 8(10):1–17. 10.2196/18160 10.2196/18160 PMC7573704 33016886

[33] Brewster PWH, Rush J, Ozen L, Vendittelli R, Hofer SM (2021) Feasibility and psychometric integrity of mobile phone-based intensive measurement of cognition in older adults. Exp Aging Res 47(4):303–321. 10.1080/0361073X.2021.1894072 33648422 10.1080/0361073X.2021.1894072 PMC8225552

[34] Wiley K, Berger P, Achim Friehs M, Lee Mandryk R (2024) Measuring the reliability of a gamified stroop task: quantitative experiment. JMIR Serious Games, 10.2196/50315 10.2196/50315 PMC11043929 38598265

[35] Staffaroni AM, Clark AL, Taylor JC et al (2024) Reliability and validity of smartphone cognitive testing for frontotemporal lobar degeneration. JAMA Netw Open. 10.1001/jamanetworkopen.2024.4266 38558141 10.1001/jamanetworkopen.2024.4266 PMC10985553

[36] Woelfle T, Pless S, Wiencierz A, Kappos L, Naegelin Y, Lorscheider J (2021) Practice effects of mobile tests of cognition, dexterity, and mobility on patients with multiple sclerosis: data analysis of a smartphone-based observational study. J Med Internet Res 23(11):1–16. 10.2196/30394 10.2196/30394 PMC8663564 34792480

[37] Haith AM, Krakauer J (2018) The multiple effects of practice: skill, habit and reduced cognitive load. Curr Opin Behav Sci 20:196–201. 10.1016/j.cobeha.2018.01.015 30944847 10.1016/j.cobeha.2018.01.015 PMC6443249

[38] De Giglio L, De Luca F, Prosperini L et al (2015) A low-cost cognitive rehabilitation with a commercial video game improves sustained attention and executive functions in multiple sclerosis: A pilot study. Neurorehabil Neural Repair 29(5):453–461. 10.1177/1545968314554623 25398725 10.1177/1545968314554623

[39] Schättin A, Häfliger S, Meyer A et al (2021) Design and evaluation of user-centered exergames for patients with multiple sclerosis: Multilevel usability and feasibility studies. JMIR Serious Games 9(2):1–25. 10.2196/22826 10.2196/22826 PMC8140386 33960956

