# Supplementary material for: CoGames: Development of an adaptive smartphone-based and gamified monitoring tool for cognitive function in Multiple Sclerosis
Source: J Neurol. 2025 Jan 15;272(2):119. doi: 10.1007/s00415-024-12818-y (PMC11735570; doi:10.1007/s00415-024-12818-y)
Supplement: Supplementary file 1 — Supplementary file1 (DOCX 13 kb) [file 415_2024_12818_MOESM1_ESM.docx]

Onlince Resource 1:

**Article title**: CoGames – Development of an adaptive smartphone-based and gamified monitoring tool for cognitive function in Multiple Sclerosis

**Journal**: Journal of Neurology

**Authors**: Silvan Pless, Tim Woelfle, Johannes Lorscheider, Andrea Wiencierz, Óscar Reyes, Carlos Luque, Pasquale Calabrese, Cristina Granziera, Ludwig Kappos

**Affiliation corresponding author**: Research Center for Clinical Neuroimmunology and Neuroscience Basel (RC2NB), University Hospital Basel, University of Basel, Switzerland

**E-mail corresponding author**: silvan.pless@usb.ch

**S1. Acceptance questionnaire**

Translation of the questions (5-point Likert scale):

**1. Enjoyment**: “To play the game X was generally…”

(1) Not enjoyable at all – (5) Very enjoyable

**2. Representation of mental function:** “I believe the game X can represent my mental performance…”

(1) Not at all – (5) Very well

**3. Adequacy of difficulty increase over the study duration:** “The difficulty increase of the game X is…” (Please ignore the repetitions of level “*Beginner*” on day 2, 6, and 11)

(1) Bad (non-gradual difficulty increase) – (5) Very good (gradually increasing difficulty)

**4. Clarity of instructions**: “The instructions of the game X were…”

(1) Not clear at all – (5) absolutely clear

**5. Frustration**: “The game X was… for me”

(1) very frustrating – (2) not at all frustrating
